# 292. Antibiotic Use Is Increased in Patients with Acute Respiratory Distress Syndrome (ARDS) Requiring Extracorporeal Membrane Oxygenation (ECMO) due to COVID-19 Compared with Influenza

**DOI:** 10.1093/ofid/ofab466.494

**Published:** 2021-12-04

**Authors:** J Alex Viehman, Christina K Thorngren, M Hong Nguyen, Palash Samanta, Cornelius J Clancy, Holt Murray, Ryan Rivosecchi

**Affiliations:** 1 University of Pittsburgh, Pittsburgh, Pennsylvania; 2 UPMC Presbyterian Hospital, Pittsburgh, Pennsylvania; 3 University of Pittsburgh Medical Center, Pittsburgh, Pennsylvania

## Abstract

**Background:**

During the COVID-19 pandemic, >50% of hospitalized patients (pts) received an antimicrobial. *E*CMO is increasingly used in COVID-19 pts with severe ARDS. ECMO has been used for ARDS due to influenza at our center in prior years. Pts on ECMO are at high risk for infections. We compared the rates of antibiotic (Ab) and antifungal (AF) use in pts on ECMO for COVID-19 vs influenza ARDS.

**Methods:**

This was a retrospective review of pts on ECMO for COVID-19 (2020-2021) or influenza (2013-2019). Antimicrobials (Abs and AFs) were categorized as anti-MRSA, anti-pseudomonal β-lactams (AP-BL), carbapenems, and new broader spectrum β-lactams. We calculated total Ab and AF utilization, adjusted for ECMO duration.

**Results:**

Seventy-one pts (36 COVID-19 and 35 influenza) were included. COVID-19 pts had longer ECMO duration (median: 25 vs 11 days, p=.03). 100% and 97% of pts with COVID-19 and influenza received ≥1 Ab, respectively, and 42% and 33% an AF, respectively. COVID-19 pts received longer duration of Abs (26 vs 10 days, p< 0.001) and but not AF. COVID-19 group (gp) were more likely to receive anti-MRSA Ab (69% vs 33%, p=.004); otherwise, there were no differences between gps in types of Abs used. When adjusted for ECMO days, COVID-19 gp received higher median number of Abs (1.23 vs 1, p=.06). Specifically, COVID-19 gp received higher median number of anti-MRSA Ab (0.2 vs 0, p=.007) and AP-BL (0.44 vs 0.28, p=.08). There was no difference in Ab-free days between gps, though the proportion of Ab-free days was lower (0.2 vs 0.36) in COVID-19 pts (p=.08). More COVID-19 pts had pathogens recovered from clinical cultures, especially S. aureus and Enterobacterales (Figure).

Pathogens recovered from clinical cultures

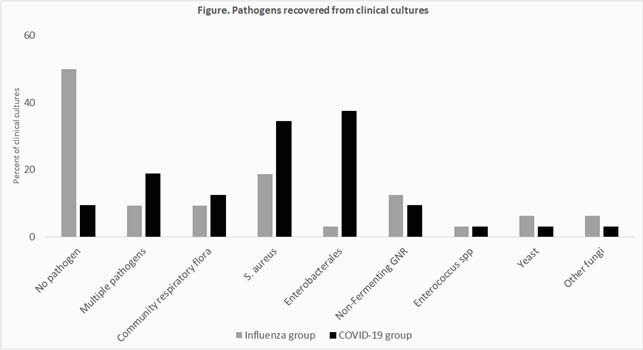

Patients recovered from clinical cultures of patients with COVID-19 and Influenza ARDS requiring ECMO

**Conclusion:**

Among pts on ECMO, those with COVID-19 received significantly longer courses of Abs than those with influenza, even after adjusting for longer durations of ECMO. Differences were driven by receipt of anti-MRSA and AP-BLs. Recovery of pathogenic bacteria was greater in COVID-19 pts than influenza pts. Given difficulties in distinguishing pneumonia from airway colonization among ARDS pts on ECMO, development of diagnostic criteria for pt care, rational antimicrobial stewardship and further research are needed.

**Disclosures:**

**Cornelius J. Clancy, MD**, **Merck** (Grant/Research Support)

